# Teacher Online Informal Learning as a Means to Innovative Teaching During Home Quarantine in the COVID-19 Pandemic

**DOI:** 10.3389/fpsyg.2021.596582

**Published:** 2021-06-24

**Authors:** Haiqin Yu, Ping Liu, Xiaoqing Huang, Yuxi Cao

**Affiliations:** School of Education, Huazhong University of Science & Technology, Wuhan, China

**Keywords:** informal learning, innovative teaching, personal teaching efficacy, general teaching efficacy, ICT efficacy, faculty, online teaching and learning

## Abstract

The home quarantine in the COVID-19 pandemic has created challenges for teaching across the world and called for innovative teaching, as well as teachers' learning. Given the rapid development of teachers' online learning and teaching, identifying effective ways to facilitate innovative teaching under such challenging conditions is a critical issue. Although researchers have realized that workplace informal learning (IL) increasingly reveals its potential value to individual development, the relationship between IL and innovation has been under-explored. The purpose of this study was to evaluate the impact of IL on innovative teaching, through the mediating roles of three types of teaching-related efficacy, with a particular focus on college teachers and online context. A sample of 479 Chinese college teachers was randomly selected to participate in the survey. The results showed that teachers' online IL in pandemic improved their personal teaching efficacy and ICT efficacy (information and communication technology efficacy), and then facilitated their innovative teaching without differences of gender and teaching-age effect. Whereas, general teaching efficacy was not a mediator between online IL and innovative teaching. Hence, we proposed a can-do motivating model of teacher efficacy in fostering innovative teaching through informal learning. It implies three properties of teachers' online IL: social interaction, autonomous learning and novelty-seeking. It also revealed that innovative teaching can be driven in COVID-19 pandemic, mainly by learning domain-specific knowledge and skills, thus enhancing personal teaching efficacy and ICT efficacy in online teaching context.

## Introduction

Cultivating innovative students is inseparable from teachers' innovative teaching, and motivating teachers to innovate is an important research issue in the field of educational innovations (Lin and Yu, 2001; Brouwer and Korthagen, 2005). Innovative teaching is supported by the increasing numbers of policies and government-funded projects designed to empower teachers in many countries (Craft, 2003). But creating conditions that allow teachers to foster innovation is a key challenge, owing to the complex and difficult transformation between teacher learning and innovation. The COVID-19 pandemic offers a particularly timely context in which to examine these issues.

Home quarantine is the main containment measure in pandemic, which has changed the daily lives of billions of people, and most work and study activities have been suspended or converted to online forms (Pellegrini et al., 2020). Hence, large-scale and prolonged online teaching is brought out. To cope with the urgent online practice, the OECD (Organization for Economic Cooperation and Development) and some countries called for teacher innovative teaching, ICT capabilities and in-depth cooperation (OECD, 2020). For example, China's Ministry of Education required universities and colleges across the country to organize teachers to learn online teaching methods, meanwhile, to provide free and open teaching and learning resources across regions and colleges (China's Ministry of Education, 2020). As a result, teacher communities of online informal learning and innovative teaching have formed throughout the country. During the time of the investigation, Chinese college teacher online learning and teaching have lasted for half an academic year. Given the rapid development of teachers' online learning and teaching caused by the current COVID-19 pandemic, identifying ways to facilitate innovative teaching under such challenging conditions is a critical issue for educational leaders to manage.

Although previous literature suggests that informal learning (IL) plays a decisive role in teacher professional development (Kyndt et al., 2016) and it is even regarded as a core constituent of individual continued learning capacity (OECD, 2003), few studies have investigated the role of informal learning in teachers' innovation. Learning has been found to account for workplace innovation and creativity in the past 30 years (de Jong and den Hartog, 2010; Anderson et al., 2014). However, the learning pattern in these studies is basically a well-designed formal learning project with structured content, such as domain-relevant and creativity-relevant knowledge and skills (Scott et al., 2004). Online informal learning is different from formal learning. First, it has no structured content, which probably makes it difficult to aim at innovation. Second, its learning method is characterized by observation and imitation, cooperation and communication, and personal reflection, which make it a highly spontaneous and personalized form of learning. In this perspective, maybe informal learning has a natural connection with innovation that emphasizes individuality. Third, teachers' motivations engaging online informal learning in pandemic are diverse and complex, such that its learning quality is difficult to be guaranteed. Online informal learning during home quarantine belongs to novelty-seeking behavior whose relationship with innovation is obscure in literature (Costa et al., 2014; Liang et al., 2020). Novelty-seeking behavior (obtaining novel information such as browsing the Web) has been found a closer link with boredom-avoiding, but it has inconsistent relationship with creativity depending on the different types of novelty-seeking behavior. For example, cyberloafing is typically portrayed as negative behavior that leads to a loss of performance and work engagement, whereas Akar and Coskun (2020) found it has significantly positive but small relationship with creativity in prospective teachers. Some researchers have asserted that it could sometimes provide creativity and flexibility to employees if control is provided, and they called for more research into its relationship with creativity. From this perspective, the relationship between informal learning and innovative teaching is not taken for granted, and whether it affects innovative teaching is unknown. The research on their relationship will help people to clarify the nature and utilization of online informal learning in teacher innovation.

Responding to this gap in the literature, we apply self-efficacy theory (Bandura, 1997) to explain how informal learning foster innovative teaching through teacher efficacy. Online informal learning during home quarantine in the COVID-19 pandemic is of a novelty-seeking behavior including novelty input and output (Liang et al., 2020), which provides a framework to explore the relationship of online informal learning and innovative teaching. Based on self-efficacy theory, the novelty input influences self-efficacy perceptions, and then promotes interest and engagement in activities, finally leading to more novelty output. Moreover, our research compared the mediating effects of three kinds of teaching-related efficacy and found their different influence mechanisms on innovative teaching. Considering the individual differences of online behaviors, we tested the moderating effect of gender and teaching age in the mediating model.

In addition to the lack of conclusive evidence regarding the relationship between informal learning and innovative teaching, there is a gap in the literature with regard to external validity. Most research on informal learning has been conducted in employee samples and innovative teaching research is conducted in primary and secondary teacher samples. The present study will examine them in a college teacher sample. As such, our study will provide some support of these concepts' external validity. Another critical gap in the existing literature involves the lack of online setting, which is particularly suited to the examination of learning and teaching in a new era of information and technology. In sum, this study extends the innovative teaching and informal learning literature to college teachers and online contexts.

### Innovative Teaching Performance

Innovative teaching performance refers to the novel and effective behavior and performance that teachers purposely apply to the teaching content, method and student evaluation, with the goal of guiding students to explore and develop their creativity. It includes three components: innovative teaching ideation (ITI), innovative teaching action (ITA) and innovative teaching outcome (ITO). ITI represents the innovation of teaching ideas and thinking, including the idea of changing and innovating teaching, the desire to learn new teaching ideas, the positive and open attitude toward education, etc. ITA represents all kinds of new teaching methods and means used by teachers, including innovating teaching objectives, designing diversified curriculum contents, flexible teaching methods, and diversified evaluation methods. ITO represents the effectiveness of teachers in cultivating students' creativity, such as supporting and encouraging students' flexible thinking, rewarding students' creative efforts and results, and recognizing and appreciating students' creative qualities.

This concept originated from the theory of individual innovative behavior (Scott and Bruce, 1994; Janssen, 2001), which includes three components according to the stages of innovation: idea generation, idea promotion and idea realization. Scott and Bruce (1994) argued that innovation begins with creative thinking or ideas, and then individuals seek support and cooperation from the environment until they are able to apply the creative idea in practice, and then complete innovation. Therefore, individual creativity involves generation of novel and useful ideas, while innovation behavior implies turning creative ideas into tangible products, including the processes of idea promotion and implementation (Amabile et al., 1996; Yao et al., 2010). Subsequently, based on the above theory, researchers developed a three-dimensional model in the context of Chinese culture, consisting of innovative ideation, action and outcome, which were collectively called innovative performance (Han et al., 2007; Zhu and Long, 2009; Zhang and Zhang, 2012; Yao and Heng, 2013).

Innovative teaching performance integrates the prior research views of creative or innovative teaching. In the literature, there are three kinds of understanding: (1) the results-oriented perspective, which aims to cultivate students' creativity (Cropley, 1997; Soh, 2000; Zhang et al., 2008); (2) the process-oriented perspective, which is characterized as innovative teaching behaviors in terms of selection of teaching content, teaching methods and design, and evaluation (Wang et al., 2010; Cai et al., 2012; Cai and Gong, 2019); (3) the quality-oriented perspective, which is based on the creative thinking and personality of teachers, such as the wisdom to create and try new methods, abilities of innovating teacher–student interaction and technology use (Lin and Yu, 2001; Xiong et al., 2020). These understandings fundamentally align with the “3p” creativity or innovation models, that is, innovation as a product perspective, as a process perspective, and as a people-centered perspective. In performance model, innovative teaching ideation corresponds to innovative qualities (creative ideas); innovative teaching actions correspond to the innovative process (behaviors); and innovative teaching outcomes correspond to the results of student cultivation. Therefore, this model is advanced and representative in its field.

Concerning on the relationship of innovative teaching and online teaching, as well as its popularity, we want to emphasize two points that have been constantly misunderstood in research and practice. First, simply using new technology does not mean innovative teaching. Innovative teaching aims to guide and inspire students to explore knowledge and cultivate students' creativity (Lueddeke, 2003; Wang et al., 2016; Zhao and Xie, 2017). Online teaching only provides the medium and greater possibility of innovative teaching, owing to the changes of teaching-learning way. Those that cannot promote the innovative development of students are not innovative teaching, even though they are novel and effective, such as using new technologies to impart knowledge, repeatedly training the students, constraining students thinking and even encouraging surface learning. Additionally, online teaching provides a urgent and prominent context for innovative teaching. Without innovation, online teaching may become a one-man show of teachers, with low interactivity, low course satisfaction and high dropout rate (Deng and Benckendorff, 2017). In sum, innovative teaching will not happen automatically in online context and does not equal to online teaching. Second, innovative teaching is not a big or radical teaching method innovation with pedagogical theory significance, but innovatively addresses new needs and changes in teaching. For example, it can involve the improvement of novel teaching methods and strategies to make them more suitable for specific students and curriculum needs; adjusting and expanding the teaching content so that it reflects progress in disciplinary knowledge and social development needs; improving the evaluating method (Wang, 2012). Therefore, there's a broad space left for teachers' innovating. These are the reasons why we choose the concept of performance-oriented innovative teaching and focus on the dimension of innovative teaching outcome in online context.

In all, innovative teaching is to use new technology and theory to solve teaching problems in a novel way, which differs from the traditional pattern of knowledge-transfer teaching and teacher-centered teaching. It is also labeled as student-centered teaching in education field (Wang, 2012; Zhao and Xie, 2017).

### Online Informal Learning and Innovative Teaching Performance

Although workplace informal learning (IL) has attracted much research attention due to its significance for both individual and organizational development (Marsick and Watkins, 2001; Conlon, 2004; Jacobs and Park, 2009), there is no singular definition of IL or unified approach to its definition at present, largely owing to the intersecting interests, contested ideas and multiple approaches in the field (Manuti et al., 2015). However, the two properties are well rooted in the literature. One is self-directed learning, with the learning content and form determined by learners themselves (Conlon, 2004; Marsick et al., 2017; Huang et al., 2020). The other is to learn through interaction and reflection, e.g., actively seeking feedback and debriefing work experiences (Manuti et al., 2015; Louws et al., 2017). IL often occurs as people carry out their work and acquire the necessary competence and interests to meet current and future work requirements (Jacobs and Park, 2009). IL mainly consists of four types: knowledge exchange, self-experimentation, environmental scanning and reflection (Lohman, 2005; Bakkenes et al., 2010; Choi and Jacobs, 2011; Huang et al., 2020). From this perspective, community-based online learning at home during the COVID-19 pandemic can be regarded as a typical form of IL because teachers opted in to open online learning resources and learning is in the hands of the teachers themselves. It is experiential learning, involving new experiences of online teaching and vicarious learning behaviors, e.g., intentionally observing others and talking with them about their work (Manuti et al., 2015; Wolfson et al., 2018). The differences between IL and online IL are the degree of individual autonomy, convenience and diversity, and evidently, online IL has more advantages. Therefore, it can better reflect the nature of informal learning. We conceptualize faculty IL as the spontaneous engagement in online learning activity organized by institutions or faculty themselves, which permeates the daily lives of teachers. In this study, IL activities include two types: (1) IL actively organized and initiated by teachers themselves, i.e., teaching exchanges in reading clubs, talks on professional development, teaching presentation; (2) IL organized by an alliance of higher education institutes and pioneer teachers, i.e., seminars or salons of teaching practice and reflection, etc., which take place beyond the college organization with inter-college and inter-disciplinary characteristics.

Research on workplace IL has mainly explored its impact on job performance, work attitude (job satisfaction, commitment), and new employees' organizational adaptation; while, there is a lack of direct research on the relation of IL and innovation. However, the relationship between some specific characteristics of IL and innovation has been verified. Previous studies reveal that IL plays a key role in fostering innovation and creativity at least for three reasons: (1) Knowledge exchange (feedback, communication and interaction) can help employees expand the space for solving innovative problems (Harrison and Rouse, 2015). (2) By self-experimentation and reflection, individuals can gain direct innovation experience and domain skills. For example, Abecassis-Moedas et al. (2016) found that entrepreneurs can learn to innovate by imitating and observing their parents, professors, colleagues or mentors, and practice immediately, thereby promoting their own level of innovation. (3) Environmental scanning can help individuals get unexpected ideas and increase flexibility (Akar and Coskun, 2020), as well as avoid costly mistakes and searching work (Gino et al., 2010). Based on the above results, IL may have a positive effect on innovation in two perspectives: interaction with others/environment and autonomous experimentation. Hence, we propose the following hypothesis:

Hypothesis 1: Teacher online IL in COVID-19 pandemic will increase innovative teaching performance.

### The Mediating Roles of Three Kinds of Teacher Efficacy Between Online Informal Learning and Innovative Teaching Performance

Teacher efficacy is defined as teachers' judgment of their capabilities to bring about desired outcomes of student engagement and learning, including those of students with learning difficulties or those who are unmotivated (Gibson and Dembo, 1984). In this study, it consists of three aspects: personal teaching efficacy (PTE), general teaching efficacy (GTE) and information and communication technology efficacy (ICTE). PTE refers to teacher's confidence on his/her individual ability to influence students; GTE refers to teacher's awareness of the role of education in student development; ICTE refers to teacher-perceived competencies to use ICT for teaching and learning purposes. Clearly, PTE and ICTE come from individual competence, while GTE comes from the cognition of the effectiveness of the whole educational enterprise in a certain cultural context, which is similar with one's vision or value.

Based on Bandura's theory of self-efficacy, the environment influences self-efficacy perceptions, and then promotes interest and engagement in activities (Bandura, 1997). During home quarantine in the COVID-19 pandemic, novelty-seeking behavior can be divided into “novelty input” and “novelty output” (Liang et al., 2020). The former refers to obtaining novel information such as online informal learning; the latter refers to engaging in creative behavior such as innovative teaching. Novelty input does not necessarily lead to novelty output. In many cases, the input may be ineffective, and we assume that the improvement of efficacy may be a critical mediating condition.

Informal learning embraces a large number of practical experiences, success stories of others and peer exchanges, which are expected to provide four sources of efficacy: mastery experiences, physiological and emotional arousal, vicarious experience and social persuasion (Bandura, 1997). Studies have found that IL can directly improve employees' and teachers' knowledge, skills and experience (Rowden and Conine, 2005; McCormack et al., 2006; Henze et al., 2009; Tannenbaum et al., 2010). For instance, workplace IL improves teacher's work-related roles and tasks, as well as changes their (often conservative) beliefs and conceptions about teaching (Kyndt et al., 2016; Louws et al., 2017). Hence, IL may increase individual teaching ability to influence students (PTE and ICTE), as well as the general views and beliefs on education (GTE). But direct research results in this field are lacking.

Although a large amount of research demonstrates that self-efficacy can predict employees' innovative behavior (de Jong and den Hartog, 2010), the research related to the impact of teaching efficacy on innovative teaching is very limited. Several preliminary studies showed that PTE can predict the innovative teaching (Wang et al., 2010; Zhang and Zhang, 2012; Cai and Gong, 2019; Xiong et al., 2020), but the relationship between GTE and innovative teaching is divergent (Wang et al., 2010; Xiong et al., 2020). Concerning ICTE, empirical research on its relation with innovative teaching is insufficient, either (Cofriyanti and Hidayanto, 2013; Yunis et al., 2018). A few studies reveal that ICTE is significantly related to ICT use (Aesaert et al., 2017), while ICT use can predict organizational performance and innovation (Cofriyanti and Hidayanto, 2013; Yunis et al., 2018). Clearly, there is no evidence of a relationship between ICTE and individual innovation. Given that the three types of teacher efficacy have different originates and prior research reveals divergent conclusions or lagging progress, it's necessary to test them respectively so as to identify the different relating routes of IL. According to the above analysis, we offer the following hypothesis:

Hypothesis 2a: PTE will significantly mediate the relationship between teacher online IL and innovative teaching performance.
Hypothesis 2b: GTE will significantly mediate the relationship between teacher online IL and innovative teaching performance.
Hypothesis 2c: ICTE will significantly mediate the relationship between teacher online IL and innovative teaching performance.

### The Moderating Effect of Gender and Teaching Age Between Online Informal Learning and Teacher Efficacy

However, engaging online IL to improve teaching efficacy is not always effective. There are gender and teaching age differences in teachers' online learning needs, teaching competence and network use efficacy (Goodyear et al., 2001; Guasch et al., 2010), which may lead to the relationship between informal learning and teacher efficacy being moderated by gender and teaching age.

For instance, female teachers have lower efficacy in internet operation and output than male teachers, while female teachers have higher efficacy in internet search and communication (Wang, 2012). In terms of training need, research shows that female teachers have greater demand for training of online teaching, but they hold more negative attitudes and face more difficulties (Wu et al., 2020). On the other hand, female teachers are more concerned with technology-integrated pedagogical knowledge, while male teachers are more concerned with pedagogical knowledge (Song et al., 2020). Junior teachers are more concerned with online teaching, and have higher efficacy in internet operation and output than senior teachers **(**Wang, 2012). These results indicate that the same training and learning may have different effects on different individuals, which may be caused by different preferences and sensitivity in terms of gender and teaching age (Tsai and Tsai, 2010; Holmes, 2013). This paper focuses on the effect of individual differences. We speculate:

Hypothesis 3a: Gender moderates the relationship between online IL and teacher efficacy during COVID-19 pandemic in that the relationship between these variables is weaker among male teachers.
Hypothesis 3b: Teaching age moderates the relationship between online IL and teacher efficacy during COVID-19 pandemic in that the relationship between these variables is weaker among senior teachers.

The current study aims to investigate whether teachers' online IL and innovative teaching are related during home quarantine in COVID-19 pandemic, whether online IL positively associates with innovative teaching through the improvement of teacher efficacy, and whether such associations are moderated by gender and teaching age differences.

**Figure 1:**
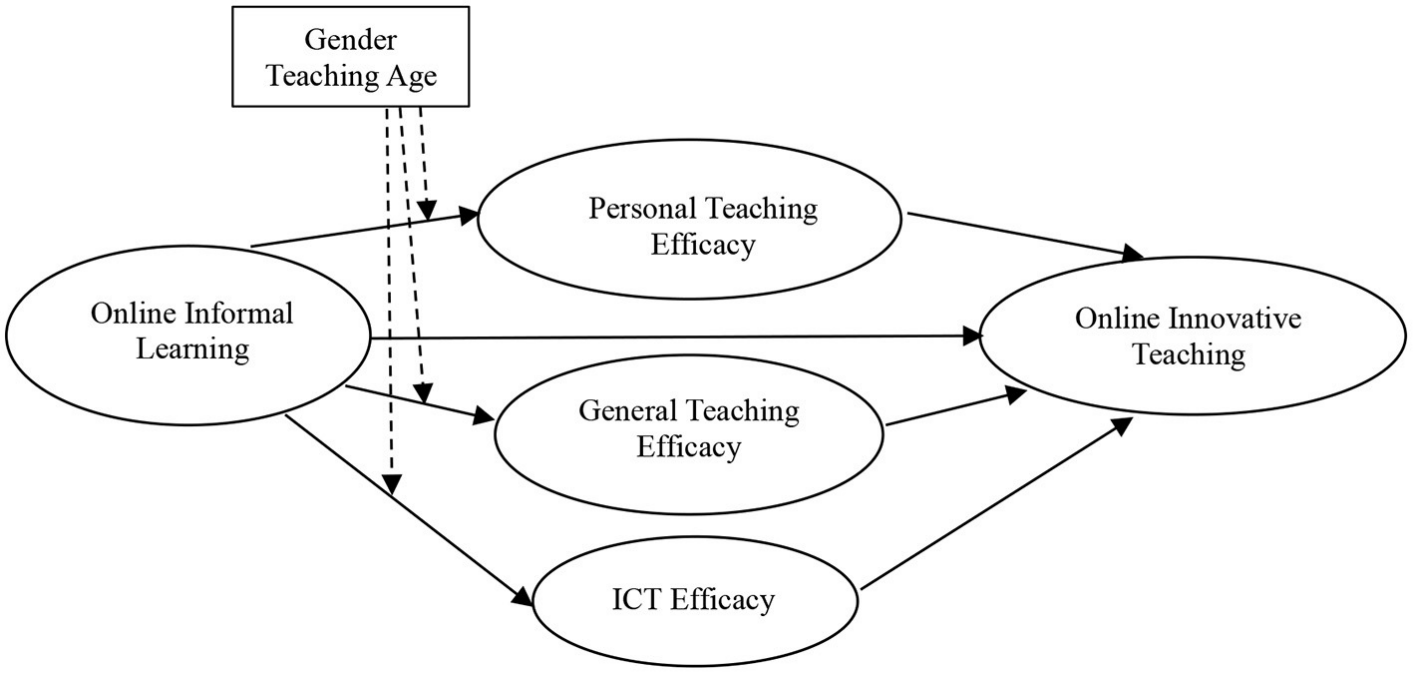
Conceptual model of the relationships tested in the study.

Our proposed model appears in Figure 1.

## Methods

### Participants and Procedure

This study aims to explore the relationship between informal learning and innovative teaching, with a mediating role of teacher's efficacy in a context of college online learning and teaching in the COVID-19 Crisis. We adopt quantitative methods to test the hypothesized relationships mentioned above. A total of 479 college teachers in China were investigated in July 2020. During the epidemic, China's Ministry of Education required universities across the country to carry out large-scale online teaching and timely organize various online training activities (China's Ministry of Education, 2020). In the context of these open learning resources and innovative teaching, the sample of this study were randomly selected from three teaching faculty alliances. With the help of group staff, the researchers asked faculty to fill out an electronic questionnaire voluntarily.

The participants consisted of 182 males (38.0%) and 297 females (62.0%). In terms of teaching age, 134 (28%) had taught for 10 years or less, 200 (41.8%) for 11–20 years, 145 (30.3%) for 21 years or more. Concerning their discipline background, 114 (23.8%) teachers taught science, 145 (30.3%) teachers taught engineering, and 220 (45.9%) teachers taught humanities and social sciences. Specifically, there were 48 (17.5%) teachers from key research-oriented institutions, 292 (61.0%) from teaching-oriented provincial institutions and 103 (21.5%) from vocational institutions. Concerning academic background, 75 (15.7%) teachers have a bachelor degree, 240 (50.1%) teachers have a master degree, 161 (33.6%) teachers have a doctoral degree, and 3 teachers have not reported their academic qualifications.

### Measures

A questionnaire consisting of three scales was used in this study, namely, Informal Learning Scale (ILS), Teacher's Efficacy Scale (TES), and the Innovative Teaching Performance Scale (ITPS). The instructions of the questionnaire asked faculty to fill in based on their learning and teaching experience over the previous five-six months of the epidemic (February–July, 2020).

The frequency of college faculty engagement in online IL activities was measured using the Informal Learning Scale (ILS), which comprised four types of IL (knowledge exchange, self-experimentation, environmental scanning and reflection). It comes from IL scales about primary and middle school teachers (Bakkenes et al., 2010; Huang et al., 2020), which is revised and extended to align with the context of this study by the researchers. The scale contains eight items. Responses were given on a 5-point Likert scale ranging from “basically no” (1) to “often” (5). Sample items include “observing and exchanging teaching techniques and methods”; “reflecting and experimenting specific teaching pattern (flipped classroom, PBL teaching, etc.)”; “participating in seminar on specific teaching problems (e.g., how to instruct students' cooperative learning and evaluate them in the context of online teaching)”; “seeking teaching resources (video conference/seminar, reading, etc.).”

The TES was adapted from Yu et al. (1995) and Moreira-Fontán et al. (2019), containing three subscales: personal teaching efficacy (PTE, six items), general teaching efficacy (GTE, six items) and ICT efficacy (ICTE, four items). Teachers rated each item on a five-point Likert scale ranging from “totally inconsistent” to “totally consistent.” Some items of this scale were as follows: (1) I have confidence in my ability to solve teaching problems (PTE); (2) In terms of teaching, I have my own set of effective methods (PTE); (3) Teachers have less influence on students than parents and society (GTE); (4) I have effectively used some special methods and techniques to deal with online teaching (ICTE); (5) I don't feel good about online teaching, and I am a little confused (ICTE).

The 16-item ITPS was adapted from Teacher Innovative Work Behavior Questionnaire (Zhang and Zhang, 2012). The scale has three dimensions: innovative teaching ideation (ITI, five items), innovative teaching action (ITA, six items), and innovative teaching outcome (ITO, five items). Participants rated each item on a five-point scale from “totally inconsistent” to “totally consistent.” Some items of this scale were as follows: (1) I often have ideas and thoughts for reforming and innovative teaching (ideation); (2) I actively organize teaching activities to enhance students interest (action); (3) I once encouraged students to propose new solutions to problems (action); (4) In my lectures, students have made innovative achievements (reports, products, programs, activities) (outcome).

### Data Analysis Strategy

All data were analyzed using SPSS 22.0 and Amos 24.0. First, a confirmatory factor analysis (CFA) was conducted to examine the construct validity for scales by Amos 24.0. Second, the descriptive statistics (M and SD) and Pearson's correlations between variables were calculated using SPSS. Third, an exploratory factor analysis (EFA) was conducted to test common method variance by SPSS. Finally, we used the bootstrapping method (5,000 bootstrap samples) to analyze the mediating role of teacher efficacy between informal learning and innovative teaching. In terms of interpreting the results, if the 95% confidence interval (CI) does not include zero, the model indicates a statistically significant mediation effect (Hayes, 2012). Additionally, multi-group SEM analysis was utilized to examine the moderating role of gender and teaching age.

## Results

### Reliability and Construct Validity of the Scales

The descriptive statistics and the correlation results of the five factors are displayed in Table 1. All five variables positively correlated with each other. As shown in Table 1, the mean of the variables ranged from 2.56 to 4.47, the standard deviation ranged from 0.50 to 0.88. The coefficients of Cronbach's α for the research variables ranged from 0.70 to 0.89, greater than the threshold of 0.7 (Wu, 2000).

**Table 1:** Descriptive statistics, Cronbach's α, and correlation matrix.

	**1**	**2**	**3**	**4**	**5**
1. IL	–	–	–	–	–
2. PTE	0.44^**^	–	–	–	–
3. GTE	0.51^**^	0.29^*^	–	–	–
4. ICTE	0.33^**^	0.60^**^	0.36^**^	–	–
5. ITP	0.44^**^	0.53^**^	0.23^*^	0.39^**^	–
*M*	2.56	4.22	3.57	3.99	4.47
*SD*	0.65	0.59	0.88	0.67	0.50
Cronbach's *alpha*	0.88	0.89	0.80	0.75	0.70

* *p < 0.05*,

** *p < 0.01. IL, informal learning; PTE, personal teaching efficacy; GTE, general teaching efficacy; ICTE, ICT self-efficacy; ITP, innovative teaching performance*.

Based on the two-step procedure recommended by Anderson and Gerbing (1988), we first performed a CFA to test the fitness of the measurement model to the research data before examining the structural relationships among the study variables. The measurement model in this study comprised five latent constructs and 27 observed indicators (three factors of Innovative Teaching and 24 items of other scales). In the CFA, latent constructs were allowed to be freely correlated with each other, and observed indicators were specified to load only on their respective latent constructs. The results of the CFA showed that the measurement model fit the data well (χ^2^ = 211.380; df = 72; χ^2^/df = 2.936; CFI = 0.962; GFI = 0.940; TLI = 0.951; IFI = 0.962; SRMR = 0.040; RMSEA = 0.047 [90% CI: 0.037, 0.056]).

We further tested the fitness of two alternative models, including a two-factor model (indicators of informal learning and teacher efficacy were loaded together on one latent construct) and a one-factor model (all 26 indicators were loaded together on one latent construct). The results of the CFA for the two-factor model were as follows: χ^2^ = 411.807, df = 73; χ^2^/df = 5.641; CFI = 0.907; GFI = 0.890; TLI = 0.884; SRMR = 0.0752; RMSEA = 0.099[90% CI: 0.089, 0.108]. The CFA results for the one-factor model were as follows: χ^2^ = 1007.585; df = 75; χ^2^/df = 13.434; CFI = 0.743; GFI = 0.688; TLI = 0.688; SRMR = 0.0973; RMSEA = 0.161 [90% CI 0.153, 0.170]. The fit index of both alternative models failed to meet the recommended criteria (Wu, 2009). The results of the chi-square statistic also demonstrated that the measurement model fit the data better than did the two-factor model (Δχ^2^ = 200.427, Δdf = 1, *p* < 0.001) or the one-factor model (Δχ^2^ = 796.205, Δdf = 3, *p* < 0.001).

### Common Method Variance Test

We used Harman's single factor analysis to test the common method variance. The results indicated that the first common factor explained only 33.68% (lower than 40%) of the total variance. There are seven factors with eigenvalues greater than one. Therefore, common method bias was unlikely to be concerned in this study.

### Hypotheses Testing

**Figure 2:**
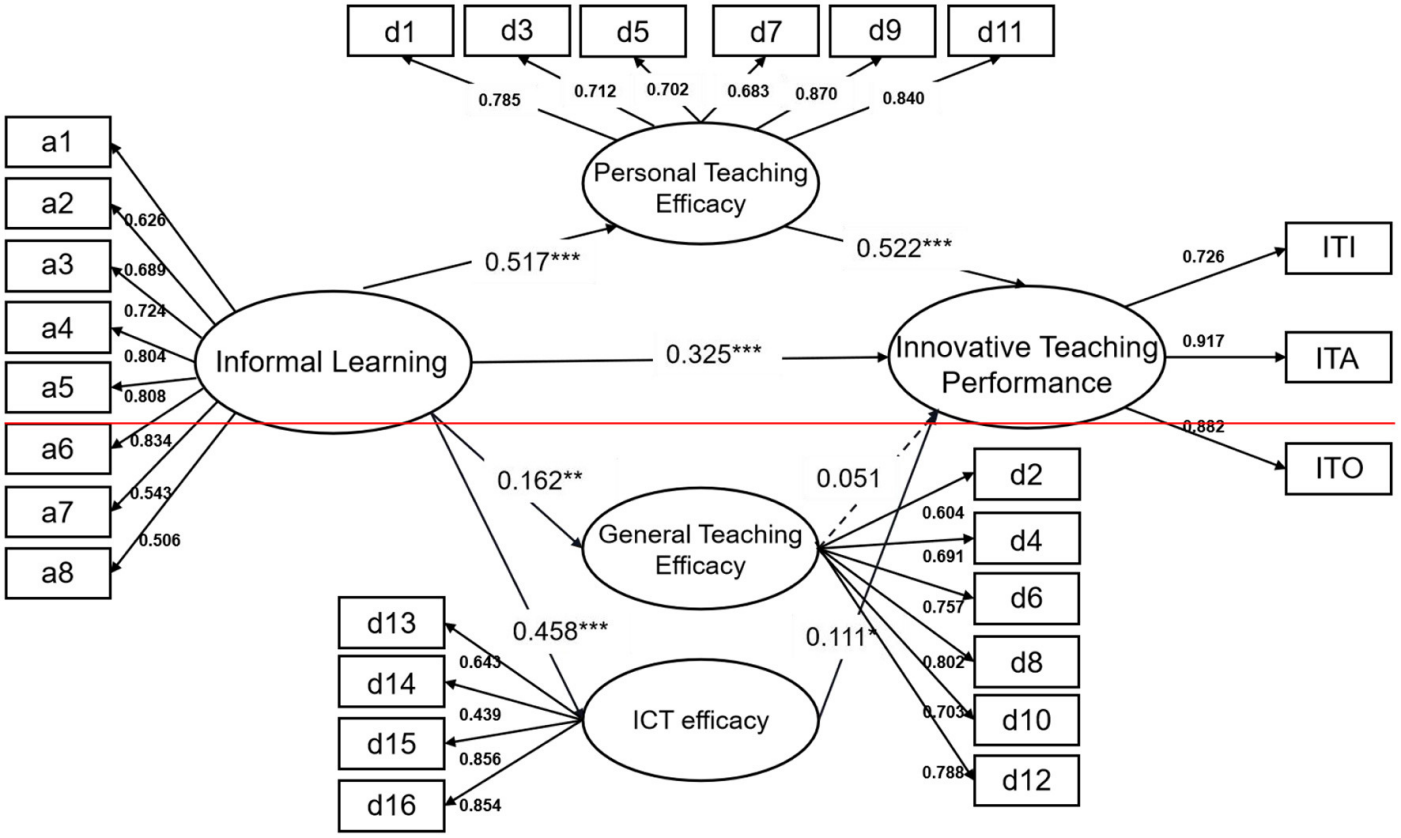
Results of SEM analysis. All the path coefficients were standardized. *N* = 479. *p < 0.05; **p < 0.01; ***p < 0.001.

We created the SEM with the Informal Learning, Personal Teaching Efficacy, General Teaching Efficacy, ICT Efficacy and Innovative Teaching Performance as the latent variables to test the proposed relationships among the study constructs. The analysis revealed that the hypothesized model demonstrated good fit to the data: χ^2^ = 773.589; df = 259; χ^2^/df = 2.987; GFI = 0.908; CFI = 0.923; IFI = 0.924; TLI = 0.911; SRMR = 0.040; RMSEA = 0.049 [90% CI 0.039, 0.057]. Next, the statistical significance of the coefficients of the direct paths among the constructs was examined. As demonstrated in Figure 2, except for the path from General Teaching Efficacy to Innovative Teaching Performance, the other hypothesized paths were statistically significant and in the expected directions. First, the direct effect of Informal Learning on Personal Teaching Efficacy, General Teaching Efficacy and ICT Efficacy were statistically significant (β = 0.517, *p* < 0.001; β = 0.162, *p* < 0.01; β= 0.458, *p* < 0.001).Second, the direct effect of Personal Teaching Efficacy and ICT Efficacy on Innovative Teaching Performance was significant(β = 0.522, *p* < 0.001; β = 0.111, *p* < 0.05). Third, the direct effect of General Teaching Efficacy on Innovative Teaching Performance was not significant (β = 0.051, *p* >0.05). Finally, the direct effect of Informal Learning on Innovative Teaching Performance was significant (β = 0.325, *p* < 0.001).

Bootstrapping analysis was performed to rigorously test the indirect relationships existing in the hypothesized model (Table 2). The results show that when personal teaching efficacy and ICT efficacy are used as mediating variables, both direct and indirect effects of informal learning on innovative teaching performance are significant, indicating that personal teaching efficacy and ICT efficacy partially mediate the impact of informal learning on innovative teaching performance. Thus, H1, H2a, and H2c are verified. But when general teaching efficacy is used as a mediating variable, the indirect effect of informal learning on innovative teaching performance is not significant, that is, general teaching efficacy has no mediating effect. H2b is not supported.

**Table 2:** Indirect effects test using bootstrapping and 95% confidence intervals (CI) for the final mediational model.

**Paths between variables**	**Bootstrapping**		**95% CI**	
	**β**	**SEB**	**Lower**	**Upper**
Informal Learning → Personal teaching efficacy → Innovative teaching performance	0.270^a^	0.034	0.208	0.343
Informal Learning → General teaching efficacy → Innovative Teaching Performance	0.008	0.007	−0.002	0.028
Informal Learning → ICT Efficacy → Innovative teaching performance	0.051^a^	0.025	0.005	0.104

a *Empirical 95% confidence interval does not overlap with 0; β = standardized coefficients; SEB, bootstrapped standard error; 95% CI, 95% confidence interval*.

A series of multigroup SEM analyses were applied to test the moderator effects in the proposed structural model. The sample was divided into two subgroups according to gender and teaching age. We used a chi-square statistic to compare a model in which all hypothesized paths were constrained to be equal across the two subgroups (i.e., constrained model) with a model in which the hypothesized path was freely estimated across the groups (i.e., unconstrained model). If the constrained model were to demonstrate a significantly higher chi-square value than the unconstrained model, this would indicate a potential moderating effect (Wu, 2013). Table 3 shows that the chi-square difference between the constrained model and the unconstrained model for two moderators was not significant, rejecting the moderating effects of gender and teaching age on the relationships between informal learning and innovative teaching performance.

**Table 3:** Chi-square difference between the constrained model and the unconstrained model.

**Moderator**	**Model**	**χ2(df)**	**χ2/df**	**Δχ2(Δdf)**
Gender	Unconstrained model	1139.345 (529)	2.154	3.42(7)
	Constrained model	1135.925 (522)	2.176	
Teaching age	Unconstrained model	1438.700 (783)	1.837	19.609(14)
	Constrained model	1458.309 (797)	1.830	

## Discussion

The COVID-19 pandemic has created challenges for online teaching across the world. But the results of this study show that crises can facilitate teachers' efforts toward innovation in online teaching under certain conditions. In general, we found that teachers' online IL in pandemic fosters their innovative teaching through the improvement of personal teaching efficacy and ICT efficacy, without differences of gender and teaching-age effect.

Our study contributes to the link of IL and innovation, providing further evidence of how “novelty input” leads to “novelty output” in the perspective of self-efficacy. We respond to Akar and Coskun (2020)'s call for more research on the relationship of online novelty-seeking behavior and creativity. Most prior research has been conducted on novelty-seeking behaviors, such as general browsing on web and cyberloafing, etc. The current study offers evidence that the online informal learning affects key variables in the teaching world. We respond to Marsick and Watkins (2001) and Kyndt et al. (2016) call for more research on how workplace informal learning (IL) link to individual development. Prior research has been conducted in employee samples and offline context, additionally, without concerns of individual innovative development. The current study offers evidence that IL fosters innovation of college teachers in online contexts.

Our study contributes to teacher efficacy literature, by identifying three different functions of PTE, GTE and ICTE in innovative teaching. Prior research reveals divergent conclusions of GTE and a gap of ICTE study. The current study offers evidence that both ICTE and PTE play critical roles in innovative teaching, while GTE cannot. Based on this, we propose a can-do motivating model of IL to foster innovative teaching. Specifically, our findings, explanations and further suggestions are around the three aspects as below.

The direct effect result shows that teachers' online IL has a positive effect on their innovative teaching (supporting Hypothesis 1). It verifies IL's property of social construction and autonomous experimentation (Watkins and Marsick, 1992; Marsick et al., 2017). Moreover, it reinforces the two streams of innovation antecedents in previous findings, that is, social interaction (Wenger and Snyder, 2000; Zhou and Lu, 2009; Liu et al., 2016) and autonomy support can improve innovation (Deci et al., 2001; Zhang et al., 2010). Social interaction with others and environment can improve individual innovation, by avoiding costly mistakes and work-searching, as well as by getting interactive feedback and alternative learning experience (Gino et al., 2010; De Stobbeleir et al., 2011; Harrison and Rouse, 2015; Abecassis-Moedas et al., 2016). Autonomy support such as choice opportunity and democratic participation in work, can make individuals feel self-determined, which will enhance their autonomous motivation, thereby fostering innovation (Zhang et al., 2010; Cai and Gong, 2019). Additionally, this study verifies a third property of IL: novelty-seeking. During home quarantine in the COVID-19 Pandemic, the Chinese government and institutions organized lots of learning resources that are more challenging and innovative than ever before. For example, for the first time, the innovative teaching competition videos of previous years were released, and expert comments were organized for teachers to learn repeatedly. Through self-recommendation, evaluation and other methods, more innovative teaching cases of ordinary teachers were selected for exchanges, and teachers were organized to conduct online discussions on hot and difficult issues in teaching. They have the opportunity to freely display and express themselves. The participation of teachers in these activities has a strong nature of seeking novelty. In sum, we believe during home quarantine in the COVID-19 pandemic, teachers' online IL has a positive effect on their innovative teaching for three reasons: social interaction with others, autonomous learning method and novelty-seeking behaviors. In the future, research on online informal learning should be strengthened to compare the differences between the post-epidemic era and the epidemic era.

The mediating effect results show that personal teaching efficacy and ICT efficacy play positive mediating effects on the link between informal learning and innovative teaching, while general teaching efficacy isn't a mediator (supporting Hypothesis 2a and 2c; rejecting 2b). Concerning the role of personal teaching efficacy and ICT efficacy, the result verifies the increase of self-efficacy belief based on domain-specific knowledge and skills is a critical factor in innovation (Amabile et al., 1996) and is a mediator to foster innovation in training (Ginamarie et al., 2004; Scott et al., 2004). It reinforces a viewpoint of self-efficacy as “can-do” motivational force by Tierney and Farmer (2002). He proposed that self-efficacy encourages the individual to engage in creative processes and maintain their level of involvement through belief in their ability to successfully accomplish these processes. This view differs from “want-to” motivational force, which reinforces that interest and enjoyment of work propels an individual to devote his efforts to creative processes (Amabile, 1993). We think the claim of “can-do” motivational role advances the interpretation of self-efficacy as domain-specific creative factor by Amabile et al. (1996), making it a theory of motivation. The current study verifies the “can-do” motivational roles of personal teaching efficacy and ICT efficacy; it implies that informal learning provides resources and capacity for innovative teaching, by empowering teachers to teach innovatively. Given that this motivational point of view hasn't got enough concern, we put forward that personal teaching efficacy and ICT efficacy play a motivating role in the COVID-19 Pandemic to foster innovative teaching. It explains why and how teacher online informal learning at home facilitate their innovative teaching.

Concerning GTE's role, the direct effect of this study shows that informal learning can predict GTE, while GTE cannot predict innovative teaching. This is different from the view of Yu and Luo (2000) on its source and nature. GTE refers to teachers' general views and judgments on the relationship between teaching and learning and the role of education in student development. Yu and Luo (2000) claim that GTE is relatively stable and does not come from personal successful teaching experience, but is affected by macro factors such as school policy, course goal tendency and national education level. This study shows that GTE can be changed by individual learning experience, however, it cannot be transformed into innovative teaching performance. We predict that GTE may take a long time to be transformed into innovation for its lack of domain-specific knowledge and skills, therefore is a remote facilitator. This study provides a negative evidence for the fuzzy relationship between GTE and innovative teaching in literature. Future research needs to further test the relationship between GTE and innovative teaching with a research design for a longer period. In all, by mediating effect analysis, this study contributes to the evidence of specific factors that motivate innovative teaching: PTE and ICTE. Discussions on the possible effects of GTE can help enrich people's understanding in this field.

The moderating effect results show that the relationship among informal learning, teacher efficacy and innovative teaching isn't influenced by gender and teaching age (rejecting 3a, 3b). We postulate that this finding might be sample-specific and context-specific. During home quarantine in the COVID-19 pandemic, teachers' online IL and innovative teaching are universally mobilized, owing to the need of dealing with boredom in a barren and under-stimulating environment. This reduces differences in people's search, use, and output of online resources. In this study, only innovative teaching and teacher efficacy have respectively difference in gender (*t* = 3.19, *p* < 0.01) or teaching age (*F* = 8.19, *p* < 0.001). Notably, in the pandemic context, teachers generally spend more time online and available free time may be a latent factor in the relationship among informal learning, teacher efficacy and innovative teaching. Hence, future research needs to rethink other individual differences such as available free time and personal traits; on the other hand, we should also note that the decrease of gender and age differences is a general trend in the use of online resources (Tsai and Tsai, 2010; Wang, 2012), which reflects the changing times and circumstances.

## Limitations, Future Research and Implications

This study has some weaknesses. First, the cross-sectional nature of the study precludes us from drawing definite causal conclusions. In addition, there is a process for the externalization of learning effects, and it may take a certain period of time to transform IL into innovation. Therefore, further studies are recommended to employ a longitudinal or mixed-method design of qualitative and quantitative methods to determine the interplay between these variables over time. Second, we used self-reports to measure variables. Self-report measures seem to be an appropriate solution to assess personal IL and efficacy, but innovation can take an objective measure, i.e., the ratings of other people (e.g., leaders or colleagues, students). Some researchers have argued that the ratings of other people may miss subtle, less visible innovative activities, capturing only those that are designed to make an impression (Purc and Laguna, 2019). It may cover the micro-level teacher–student interaction and enlightenment of students' thinking in teaching. Future research should consider such problems, and researchers can use other measures and diverse standards of innovative teaching. Third, the sample was from a single country (China) which may influence the relationships between IL and innovative teaching. Cultural differences have been considered important with respect to innovation (Rosenbusch et al., 2011). China is experiencing a wave of teaching innovation, and the country's special situation should be kept in mind. Future cross-cultural research and/or research in other cultural contexts are encouraged. Fourth, the current study is conducted during home quarantine in the COVID-19 pandemic, so that the effect of IL may be triggered for much available free time of people, and the special context should be kept in mind. In all, it needs to be furthered in a variety of different samples (employees or primary and secondary school teachers, etc.) and different situations (online context in post-pandemic, offline context, etc.).

The findings have some implications for the practice of faculty development and empowering teachers (Gaff and Simpson, 1994; Lueddeke, 2003; Farris-Berg, 2014). They broaden the content and modes of innovation intervention. First, educational institutes should treat teachers as active agents in their own development who self-direct their learning, rather than set mandatory requirements for study hours, study content and place of study. Second, innovative teaching may be developed through interventions targeted at teaching efficacy and ICT efficacy. The support for informal learning should focus on building teacher online communities (Lueddeke, 2003), providing alternative opportunity for teachers' reflective exchange of teaching experiences, and inputting enough novel and challenging teacher' learning resources, thus facilitating the transformation between input and output.

## Data Availability Statement

The original contributions presented in the study are included in the article/supplementary material, further inquiries can be directed to the corresponding author.

## Ethics Statement

The studies involving human participants were reviewed and approved by the ethics committee of Huazhong University of Science & Technology. The patients/participants provided their written informed consent to participate in this study.

## Author Contributions

HY designed the research and wrote the manuscript. PL analyzed some part of data. XH was responsible for some tables, language editing, and collating the format of the paper. YC was responsible for all literature. All authors contributed to the article and approved the submitted version.

## Conflict of Interest

The authors declare that the research was conducted in the absence of any commercial or financial relationships that could be construed as a potential conflict of interest.
